# Correlated Factors with Quitting Attempts Among Male Smokers in Vietnam: A QUITLINE-Based Survey

**DOI:** 10.3390/ijerph16010084

**Published:** 2018-12-30

**Authors:** Chau Quy Ngo, Ryan G. Chiu, Hanh Thi Chu, Giap Van Vu, Quang Nhat Nguyen, Long Hoang Nguyen, Tung Thanh Tran, Cuong Tat Nguyen, Bach Xuan Tran, Carl A. Latkin, Cyrus S.H. Ho, Roger C.M. Ho

**Affiliations:** 1 Department of Internal Medicine, Hanoi Medical University, Hanoi 100000, Vietnam; ngoquychaubmh@gmail.com (C.Q.N.); chuthihanhbmh@gmail.com (H.T.C.); vuvangiap@hmu.edu.vn (G.V.V.); 2 College of Medicine, University of Illinois at Chicago, Chicago, IL 60612, USA; rchiu6@uic.edu; 3 Center of Excellence in Health Service and System Research, Nguyen Tat Thanh University, Ho Chi Minh City 700000, Vietnam; 4 Université Claude Bernard Lyon 1, 69100 Villeurbanne, France; quang.n.nguyen@alumni.duke.edu; 5 Center of Excellence in Evidence-based Medicine, Nguyen Tat Thanh University, Ho Chi Minh City 700000, Vietnam; tung.coentt@gmail.com; 6 Center of Excellence in Behavioral Medicine, Nguyen Tat Thanh University, Ho Chi Minh City 700000, Vietnam; longnh.ph@gmail.com (L.H.N.); pcmrhcm@nus.edu.sg (R.C.M.H.); 7 Institute for Global Health Innovations, Duy Tan University, Da Nang 550000, Vietnam; cuong.ighi@gmail.com; 8 Institute for Preventive Medicine and Public Health, Hanoi Medical University, Hanoi 100000, Vietnam; 9 Johns Hopkins Bloomberg School of Public Health, Baltimore, MD 21205, USA; carl.latkin@jhu.edu; 10 Department of Psychological Medicine, National University Hospital, Singapore 119074, Singapore; cyrushosh@gmail.com; 11 Department of Psychological Medicine, Yong Loo Lin School of Medicine, National University of Singapore, Singapore 119077, Singapore

**Keywords:** smoking, quitting, attempt, quitline, Vietnam

## Abstract

Despite its decreasing prevalence, cigarette smoking remains the second leading cause of preventable death worldwide. In Vietnam, despite recent smoking cessation efforts, the prevalence of tobacco consumption remains high, particularly among males. In this study, we aim to evaluate the self-efficacy in quitting smoking (i.e., quitting confidence), intention to quit, and identifying associated factors among both rural and urban Vietnamese male populations. A cross-sectional study was conducted on 321 patients (52.7% urban and 47.4% rural inhabitants) who utilized QUITLINE services of Bach Mai Hospital (Hanoi, Vietnam). Socio-economic status, smoking history, cigarette usage data, and intent to quit were assessed. Baseline data were correlated with quitting confidence, to identify significant associated factors. The majority (75.9%) of participants were in the planning phase of cessation, yet 90.8% lacked complete confidence in their quitting ability. Older age, fewer cigarettes per day and previous quitting attempts were associated with quitting confidence (*p* < 0.05) and plans to quit (*p* < 0.05). Older smokers and previous quitters were more confident in their ability to quit in the near future and more likely to have made plans to quit. Future smoking cessation efforts should focus on improving self-efficacy, particularly among younger and newer smokers.

## 1. Introduction

Smoking remains the second leading cause of early death and disability worldwide, resulting in 10 million lives lost annually and USD 1.9 trillion in lost productivity, wages and healthcare expenditure worldwide [1,2,3,4]. It is also estimated that the age-adjusted prevalence of daily tobacco smoking is 31.1% for men and 6.2% for women [5]. Furthermore, as a global average, roughly 50% of young men and 10% of young women are projected to become smokers in their lifetime if current prevalence figures persist [3,5].

Recognizing the tremendous benefits of smoking cessation, an increasing number of national smoking cessation initiatives have been carried out including the taxation of cigarettes and other tobacco products, restrictions on their marketing and promotion, bans on their consumption in public venues, and the mandated placement of warning labels on sold items [1]. Nevertheless, the impact of such programs has been inconsistent and variable among countries [6]. In developing countries, including Vietnam, socio-economic factors and the resulting lack of access to quitlines and the availability of other cessation services, remain key obstacles to the effective promotion of smoking cessation [6]. Moreover, the lack of policy and public awareness surrounding the risks associated with cigarette use, in addition to industry influence, further impede progress [6,7,8]. It follows that even as assistance becomes more readily available in these countries, the rates of use of such services have been significantly underutilized in comparison with more developed nations [9].

In Vietnam, a series of public health reforms, including the utilization of village health workers as community health promoters and smoking cessation counselors, have contributed to the decline in tobacco use since the 1990s [10,11,12]. Of note are QUITLINE services, which are nationally standardized, locally administered, toll-free, phone-based systems for tobacco abuse education offering support for those planning to quit smoking and maintenance support for those who have already ceased smoking [10]. These programs have formed a relatively low-cost and integral part of cessation services across the country. Most, however, are currently managed separately by individual healthcare institutions and require consolidation from the Vietnamese government to equalize standards of care and information provided to clients [10]. As a result, despite the implementation of the aforementioned policies, the prevalence of smoking among Vietnamese males remains high (45.3%) [13]. Furthermore, while data are available regarding cessation and cessation attempt rates [13], studies examining potential correlates to smoking cessation intention and self-efficacy remain limited.

Self-efficacy has been proposed in some behavioral theories such as the transtheoretical model, the theory of planned behavior or the health belief model as a critical component for the success of smoking cessation. Increasing self-efficacy may motivate the initiation and maintenance of smoking cessation, as well as relapse prevention [14]. In this study, we aim to evaluate the self-efficacy in quitting smoking (i.e., quitting confidence), intention to quit and identify associated factors among both rural and urban Vietnamese male populations. Smoking prevalence among males in Vietnam is one of the highest in the world. A greater understanding of the potential correlates of smoking cessation success might provide important insights for future health policy reform and community efforts to support and sustain the impact of smoking cessation initiatives in Vietnam.

## 2. Materials and Methods

### 2.1. Study Design and Sampling

We conducted a cross-sectional study from June to July 2016 at the Respiratory Center at Bach Mai Hospital where the QUITLINE service was established in September 2015 to support tobacco users to quit tobacco use. The service, which was provided by 10 consultants certified in counseling drug treatment, is available to patients every day of the week from 8:00 a.m. to 10:00 p.m. free-of-charge.

Convenience sampling was utilized to recruit a total of 321 male participants according to the following inclusion criteria: (1) being at least 18 years of age, (2) having contacted the QUITLINE service to schedule an appointment, and (3) being available to participate in a 5- to 10-minute phone interview. All participants were informed of the study risks and benefits and the protection of their confidentiality prior to their participation in the study.

### 2.2. Measurements and Instruments

Participants contacting the QUITLINE service were initially invited to participate in the survey using a structured questionnaire before receiving counseling services. Participant information about socio-economic characteristics (age, education, employment, and living area), cigarette consumption patterns (types of cigarette and daily frequency of usage), history of smoking cessation, and motivation to quit smoking were collected via closed-ended questions.

#### 2.2.1. History of Smoking Cessation

Participants history of smoking cessation attempts were collected. This history recorded attempts to quit smoking in the past year (prior to contacting the QUITLINE service, the number of cigarettes consumed after attempting to quit and the duration of smoking abstinence if applicable.

#### 2.2.2. Motivation to Quit Smoking

Participants were asked about their plans (if any) to quit smoking in the next year. They were also asked to assess their confidence in being able to quit smoking on a scale from 0 to 10 points, with a score of 10 indicating complete confidence in one’s ability to successfully quit smoking. This scale was adapted from recommendations by the Mayo Clinic for quantifying cessation confidence [15].

### 2.3. Statistical Analysis

The data were analyzed using STATA version 12.0 (Stata Corp. LP, College Station, TX, USA). Chi-square and Mann-Whitney *U*-tests were conducted to compare socio-economic data, history of smoking cessation and motivation to quit smoking by living area (rural or urban), to determine if participants differed significantly by geographic origin, before identifying factors associated with our primary endpoints. Multivariate logistic regression was then employed to identify factors associated with a motivation to quit in the next year and complete confidence in quitting smoking, controlling for any significant baseline differences in demographic variables between rural and urban participants. A multivariate Tobit regression was used to assess factors associated with the smoking cessation confidence score. A pool of variables was used as potential associated factors with outcomes of interest, including socio-demographic characteristics (age, marital status, education, employment), smoking behaviors (type of cigarettes used, number of cigarettes use per day), and history of smoking cessation (attempts to quit smoking before contacting the QUITLINE service, attempts to quit last year). Stepwise backward strategies were combined with these regression models, using the *p*-value of log-likelihood of 0.2 as the threshold for selecting the variables, to construct the final models. Statistical significance was set at *p* < 0.05.

### 2.4. Ethics Approval

The protocol of the study was approved by the Institutional Review Board of Bach Mai Hospital.

## 3. Results

Among the 321 male participants, roughly half resided in rural areas (52.7%). Most were married (81.6%), had obtained high school education (52%), and were blue-collar workers (41.7%). Regarding cigarette usage pattern, 95% of the participants utilized standard cigarettes with the median number of daily cigarette consumption per day being 13.8 (25th–75th percentile: 7–20) (Table 1).

Among study participants, we observed that a high percentage (79.8%) had attempted to quit smoking in the last year before contacting the QUITLINE service (Table 2). After attempting to quit, 76.2% of them reported not smoking another cigarette. However, the duration of their smoking abstinence remains low at 0.27 months. The methods used to quit smoking among these participants were highly variable (i.e., non-nicotine gum, oral medications, counseling, or a combination thereof).

Table 3 illustrates the motivation to quit smoking among participants. Most of them (75.9%) reported wanting to quit smoking in the coming year. However, only 9.2% were completely confident about quitting smoking, with a mean quit smoking confidence score of 6.7 (Standard deviation (SD) = 2.3). A high proportion of participants reported having received support from relatives/friends (65.6%) or their spouses/partners (69.6%). Importantly, the most common reasons for reported smoking relapse were being around people who smoked (63.9%), and feeling uncomfortable if not smoking (55.0%).

Table 4 summarizes the factors associated with quitting confidence and planning to quit in the next year. We found that the higher the quit smoking confidence score of the participant, the more likely he/she planned to quit smoking in the coming year (Odds Ratio (OR) = 1.30, 95% Confident Interval (CI) = 1.04, 1.62). Older participants were also likely to have more confidence in quitting smoking (OR = 1.05, 95% CI = 1.02, 1.09). Importantly, the number of cigarettes per day had a negative association with the confidence of being able to quit smoking (OR = 0.94, 95% CI = 0.89, <1.00). Lastly, the participants who tried to quit smoking in the last year were more likely to have a higher quit smoking confidence score (coeficient = 0.80, 95% CI = 0.01,1.58). Marital status, educational attainment level, living area, and previous self-managed attempts to quit were not associated with one’s confidence to successfully quit or having a plan to quit smoking.

## 4. Discussion

In Vietnam, smoking cessation initiatives that have shown success in other countries, have suffered not only from a relative scarcity of funding and government support but also from a lack of consolidation around evidence-based guidelines [2,10]. In this study, we found that participants’ age and previous quit attempts were positively associated with confidence in one’s ability to cease smoking in the next year, and that conversely, the number of cigarettes one consumed per day was negatively associated with this outcome. It also follows that confidence, as assessed by the 12-point confidence scale, was positively correlated with having a plan to quit within the next year. It should be noted that although the cross-sectional nature of this study is unable to determine the order of correlated events, previous literature suggests that self-efficacy precedes intention and attempts to quit, rather than vice versa [16]. These findings may serve as a basis upon which future national standards, and additional public policy, may be better informed, through a greater focus on fostering the aforementioned sense of confidence and self-efficacy among younger and/or newer quitters.

While the association between previous cessation attempts and perceived future cessation ability is widely supported by the available literature, the positive relationship between age and quitting confidence conflicts with some reports from other countries [17]. Some of these argue that older patients are less likely to attempt to quit smoking compared to their younger counterparts, while others vice versa [18,19,20,21]. Nonetheless, this study serves as the first to evaluate the strength of age as a correlate to smoking cessation readiness in a strictly Vietnamese population.

The observed association may be due to the fact that many clinical sequelae associated with tobacco use, such as cardiovascular disease and lung cancers, do not manifest until use has become chronic [2]. It follows, that in witnessing first-hand the negative effects of smoking, patients may then become more motivated to quit as a result. This may, in turn, be compounded by the fact that information regarding the adverse effects associated with smoking have not traditionally been as widely disseminated in Vietnam compared to Western countries, where anti-smoking campaigns have long been propagated through: government policy/regulation, philanthropic endeavors, mass media, and scholastic drug abuse education programs [22]. As a result, asymptomatic Vietnamese smokers and those contemplating becoming smokers may not be as aware of the long-term adverse health outcomes compared to their Western counterparts. In addition, cultural factors particularly salient among Vietnamese males may compound these effects, as smoking has long been seen as an integral part of male social status and activity [23]. Nevertheless, the manifestation of symptoms of chronic smoking in late life, combined with increasing lifespans and an aging population, creates additional economic and healthcare burdens that should be addressed by future public policy and the adoption of evidence-based standards.

The negative association between smoking frequency (in terms of cigarettes per day) and participants’ confidence scores are not particularly surprising, as the relationship between the frequency of tobacco consumption and cessation confidence is well-documented in the literature [24,25,26,27]. Furthermore, the correlation between higher confidence in cessation and being in the planning phase of cessation also supports findings from previous studies [28,29,30,31,32]. Nevertheless, these findings also indicate the need for greater focus on identifying and encouraging those smokers who are in the earlier stages of addiction (i.e., those who may not currently smoke as many cigarettes per day as those who have a long smoking history), to quit sooner rather than later.

The implications of the results of this study on Vietnamese public health policy point to the creation of policies that foster the promotion of positive correlates in the planning phase of smoking cessation. Namely, approaches and strategies that boost quitting confidence and lower the frequency of cigarette consumption may prove fruitful in increasing quitting success rates in these populations. Furthermore, our results suggest that additional support might be needed for younger smokers and smokers with no previous history of quitting attempts, as they were found to be less likely to possess the necessary self-confidence in smoking cessation, and less likely to be in the planning phase of quitting. For example, future efforts could focus on providing active encouragement to individuals contemplating quitting, enhancing self-efficacy and boosting willpower in line with the cultural values of many Vietnamese individuals, who often cite personal will as “all it takes” to overcome addiction [33]. The behavioral support that these actions offer may bolster the positive and negative reinforcement of smoking cessation, and thus increase the rate at which quit attempts are made, and ultimately succeed [34].

### Limitations

Despite roughly equal representation of participants from both urban and rural settings, as a single-center study, the patient population eligible and available for inclusion in our study may not be fully representative of the Vietnamese population as a whole. Additionally, the entirely male population of the study serves as a limitation to the external validity of this study on the general population. However, given the stark differences in cultural expectations and behaviors between Vietnamese males and females, the results presented here are applicable to the creation of effective policies and interventions targeting males in particular, among whom smoking remains far more common than in females [23]. Moreover, the existence of stigmas surrounding smoking and its cessation may have caused either under or over self-reporting of some information in the interview [35].

Lastly, it is important to note that the participants within this study are individuals who were willing to call the QUITLINE service. Consequently, the factors correlated with smoking cessation confidence in this study may not be the same for those smokers who were not as motivated, or who are otherwise unwilling or unable to contact QUITLINE. Nonetheless, as contemplation is only one step toward actualization of smoking cessation, identifying factors associated with quitting among contemplative individuals might help us better focus resources in the development of more targeted and effective programs to decrease smoking prevalence.

## 5. Conclusions

Among Vietnamese male smokers, older age and previous attempts to quit were positively associated with confidence in one’s own ability to quit in the near future. It follows, that future policies and interventions aimed at reducing the prevalence of smoking, should be designed in order to better help younger smokers and those in the pre-contemplative phase of smoking cessation to better formulate plans to quit in the near future, and boost confidence in their ability to overcome addiction.

## Figures and Tables

**Table 1:** Socio-economic characteristic of the participants.

Characteristic	Living Area						*p*-Value
	Rural		Urban		Total		
	*n*	%	*n*	%	*n*	%	
**Total**	169	52.7	152	47.4	321	100	
**Age group (years)**							0.04
<30	37	21.9	42	27.6	79	24.6	
30–44	70	41.4	57	37.5	127	39.6	
45–59	50	29.6	31	20.4	81	25.2	
60+	12	7.1	22	14.5	34	10.6	
**Marital status**							0.38
Single	28	16.6	31	20.4	59	18.4	
Married	141	83.4	121	79.6	262	81.6	
**Education**							<0.01
Below high school	69	40.8	28	18.4	97	30.2	
High school	79	46.8	88	57.9	167	52.0	
Above high school	21	12.4	36	23.7	57	17.8	
**Employment**							<0.01
Blue-collar workers	87	51.5	47	30.9	134	41.7	
White-collar workers	21	12.4	28	18.4	49	15.3	
No income	26	15.4	39	25.7	65	20.3	
Others	35	20.7	38	25.0	73	22.7	
**Type of cigarette used**							
Standard cigarette	160	94.7	145	95.4	305	95.0	0.77
Rolling tobacco	2	1.2	3	2.0	5	1.6	0.57
Bamboo water pipe	16	9.5	19	12.5	35	10.9	0.38
**Number of cigarettes/day**							0.05
<10	51	32.1	35	24.1	86	28.3	
10–19	51	32.1	49	33.8	100	32.9	
20–29	54	34.0	49	33.8	103	33.9	
30+	3	1.9	12	8.3	15	4.9	
	**Median**	**25th–75th**	**Median**	**25th–75th**	**Median**	**25th–75th**	
**Age (years)**	40	31–49	37.5	29–51.5	39	30–50	0.73
**Number of cigarettes/day**	13	7–20	13.8	10–20	13.4	7–20	0.27

**Table 2:** History of smoking cessation.

Characteristic	Living Place						*p*-Value
	Rural		Urban		Total		
	*n*	%	*n*	%	*n*	%	
**Tried to quit smoking before contacting QUITL** **INE service**							0.62
No	36	21.3	29	19.1	65	20.3	
Yes	133	78.7	123	80.9	256	79.8	
**Attempted to quit in the last 12 months**							0.74
No	41	24.7	40	26.3	81	25.5	
Yes	125	75.3	112	73.7	237	74.5	
**Total number of cigarettes smoked after attempting to quit**							0.83
None	103	77.4	92	74.8	195	76.2	
1–5 cigarettes	16	12.0	18	14.6	34	13.3	
More than 5 cigarettes	14	10.5	13	10.6	27	10.6	
**Methods used to quit smoking**							
Direct counseling	1	0.8	2	1.6	3	1.2	0.52
Nicotine replacement therapy	2	1.5	3	2.4	5	2.0	0.59
Using non-nicotine gum	9	6.8	12	9.8	21	8.2	0.38
Use medicine (Bupropion/Varenicline)	6	4.5	6	4.9	12	4.7	0.89
Not using any method	8	6.0	6	4.9	14	5.5	0.69
Others	1	0.8	0	0.0	1	0.4	0.34
	**Median**	**25th–75th**	**Median**	**25th–75th**	**Median**	**25th–75th**	
**Duration of smoking abstinence (months)**	0.2	0.06–2.8	0.4	0.07–12	0.27	0.07–12	0.55

**Table 3:** Attempts to quit smoking.

Characteristic	Living Place						*p*-Value
	Rural		Urban		Total		
	*n*	%	*n*	%	*n*	%	
**Planning to quit in the coming years**							
No	37	21.9	40	26.7	77	24.1	0.32
Yes	132	78.1	110	73.3	242	75.9	
**Quitting confidence**							
Not completely confident	125	89.9	112	91.8	237	90.8	0.60
Completely confident	14	10.1	10	8.2	24	9.2	
**Received support from**							
Relatives, friends	85	65.9	75	65.2	160	65.6	0.91
Spouses/partners	88	68.2	81	71.1	169	69.6	0.63
**Reasons for smoking relapse**							
Feeling uncomfortable	69	53.9	64	56.1	133	55.0	0.73
People around smoking	85	66.9	69	60.5	154	63.9	0.30
Stress at work	42	32.8	51	45.1	93	38.6	0.05
Weight gain	5	3.9	3	2.6	8	3.3	0.58
Others	21	16.4	11	9.7	32	13.2	0.12
	**Mean**	**SD**	**Mean**	**SD**	**Mean**	**SD**	
**Quit smoking confidence (score)**	6.7	2.3	6.6	2.3	6.7	2.3	0.66

SD: Standard Deviation.

**Table 4:** Associated factors with quitting attempts and quitting confidence.

Characteristic	Completely Confident of Quitting		Quit Smoking Confidence (Score)		Planning to Quit in the Coming Years	
	OR	95% CI	Coef.	95% CI	OR	95% CI
**Quitting confidence (score)**					1.30 **	1.04, 1.62
**Age**	1.05 ***	1.02, 1.09	0.04 ***	0.02, 0.07		
**Marital status**						
Single	ref		ref			
Married	0.41	0.11, 1.52	−0.67	−1.53, 0.19		
**Education**						
Below high school	ref				ref	
Above high school	0.37	0.08, 1.69			4.55	0.56, 37.01
**Living area**						
Rural					ref	
Urban					0.45	0.15, 1.36
**Tried to quit smoking before contacting QUITLINE service**						
No	ref					
Yes	0.36	0.08, 1.62				
**Number of cigarettes per day**	0.94 **	0.89, <1.00	−0.03 **	−0.06, <−0.00		
**Attempted to quit last year**						
No			ref			
Yes			0.80 **	0.01, 1.58		

*** *p* < 0.01, ** *p* < 0.05.
